# Trends in canine seroprevalence to *Borrelia burgdorferi* and *Anaplasma* spp. in the eastern USA, 2010–2017

**DOI:** 10.1186/s13071-019-3735-x

**Published:** 2019-10-14

**Authors:** Bhagya Galkissa Dewage, Susan Little, Mark Payton, Melissa Beall, Jennifer Braff, Donald Szlosek, Jesse Buch, Andrew Knupp

**Affiliations:** 1 Department of Veterinary Pathobiology, Center for Veterinary Health Sciences, Oklahoma State University, Stillwater, Oklahoma USA; 2 Department of Statistics, College of Arts and Sciences, Oklahoma State University, Stillwater, Oklahoma USA; 3 IDEXX Laboratories, Inc., Westbrook, Maine USA

**Keywords:** *Anaplasma* spp., Anaplasmosis, *Borrelia burgdorferi*, Canine, Epidemiology, Lyme borreliosis, SNAP®4Dx® Plus Test

## Abstract

**Background:**

*Borrelia burgdorferi* and *Anaplasma phagocytophilum* are tick-borne infections transmitted by *Ixodes scapularis* in the eastern USA; both agents cause disease in dogs and people. To characterize changes in seroprevalence over time, Cochran Armitage trend tests were used to evaluate percent positive test results for antibodies to *B. burgdorferi* and *Anaplasma* spp. in approximately 20 million canine tests from 2010–2017 in 25 states and 905 counties in the eastern USA.

**Results:**

A significant decreasing trend in seroprevalence to *B. burgdorferi* was evident in eight states along the mid-Atlantic coast from Virginia to New Hampshire, and in Wisconsin. In contrast, a continued increasing trend was evident in five northeastern and Midwestern states where Lyme borreliosis is endemic or emerging, as well as in three southern states where endemicity has not yet been widely established. Similarly, seroprevalence to *Anaplasma* spp. showed a significant, although smaller, decreasing trend in five states along the mid-Atlantic coast from Virginia to Connecticut and Rhode Island, as well as in Minnesota and Wisconsin in the Midwest; despite the fact that those trends were significant they were weak. However, a strong increasing trend was evident in Massachusetts and three states in northern New England as well as in Pennsylvania.

**Conclusions:**

As expected, seroprevalence continued to increase in regions where Lyme borreliosis and anaplasmosis are more newly endemic. However, the declining seroprevalence evident in other areas was not anticipated. Although the reasons for the decreasing trends are not clear, our finding may reflect shifting ecologic factors that have resulted in decreased infection risk or the combined positive influence of canine vaccination, tick control, and routine testing of dogs in regions where these infections have long been endemic. Analysis of trends in canine test results for tick-borne infections continues to be a valuable tool to understand relative geographical and temporal risk for these zoonotic agents.
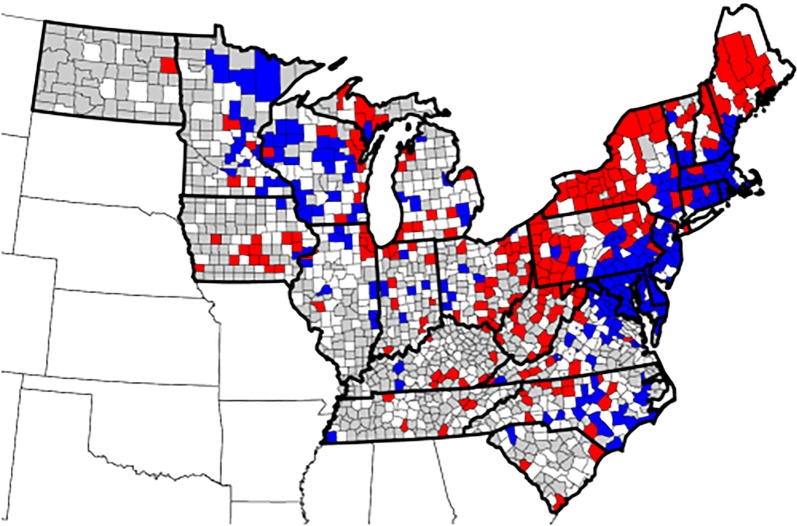

## Background

Lyme borreliosis (LB) and anaplasmosis (AN) are important vector-borne diseases of people and dogs in North America. The agents responsible for both diseases are maintained in wildlife reservoirs and transmitted by *Ixodes* spp. ticks. In people, LB caused by *Borrelia burgdorferi* (*sensu stricto*) (hereafter referred to as *Bb*) is the most commonly reported tick-borne disease in the USA; more than 40,000 new cases are reported annually, with recent estimates suggesting as many as 300,000 people per year are actually diagnosed [1]. Far fewer cases of AN caused by *Anaplasma phagocytophilum* (*Ap*) are reported to CDC. However, the annual number of reported AN cases increased from 1761 to 5762 from 2010 to 2017 [2, 3]. Although ample data are available on prevalence of canine infection by antibody testing, and *Ixodes* spp. ticks are commonly reported from dogs in this region, the incidence of clinical LB or AN in dogs is not well understood [4–7].

Humans infected with *Bb* often present with an erythema migrans rash and mild flu-like symptoms; if untreated, arthritis, carditis, or neurological disease may develop [8]. Infection with *Bb* is considered asymptomatic in many dogs, but some canine patients will develop arthritis, and, less commonly, severe, fatal glomerulonephritis [9]. People with disease due to *Ap* initially develop fever, headache, and myalgia which, if untreated, can progress to renal failure and respiratory distress [10]. Anaplasmosis became a nationally notifiable disease in the USA in 2000 and, since that time, the incidence has increased from 1.4 cases per million persons to 18.3 cases per million persons as of 2017 [3]. Canine infection with *Ap* may cause fever, thrombocytopenia, lethargy, and polyarthritis [11], and prevalence of antibodies to *Anaplasma* spp. in dogs in the northeastern USA increased from 5.5% to 7.1% between 2007 and 2012 [4, 5].

Small mammals serve as reservoir hosts for both *Bb* and *Ap*; in the eastern and midwestern US, infection is transmitted between reservoir hosts and to humans and dogs by *Ixodes scapularis* [12]. Abundant, expanding *I. scapularis* populations have resulted in increased incidence of both human and veterinary LB and AN as well as several other infections [13]. According to recent surveys on pet ownership, dogs are present in 48% of households in the USA, or approximately 60.2 million homes [14]. Several studies have documented the utility of using dogs as sentinels for human tick-borne diseases [15, 16]. Dogs receiving veterinary care are routinely tested each year for heartworm infection, and this test is often paired with screening for antibodies to *Bb* and *Anaplasma* spp., allowing evaluation of year-on-year changes in seroprevalence for these two agents. In the present study, we evaluated recent geographical trends in seroprevalence for antibodies to *Bb* and *Anaplasma* spp. in dogs in 25 states in the eastern USA to characterize changes in canine seroprevalence over the 8-year study period.

## Methods

### Source of data

The data for the present study was obtained using the SNAP® 4Dx® and SNAP® 4Dx® Plus test kits, commercial diagnostic test devices (IDEXX Laboratories, Inc., Westbrook, Maine, USA) widely used in veterinary medicine. These test kits utilize an enzyme linked immunosorbent assay for the simultaneous qualitative detection of canine antibodies against tick-borne agents, including *Borrelia burgdorferi*, *Ehrlichia canis*, *Ehrlichia ewingii* (for 4Dx® Plus), *Anaplasma phagocytophilum* and *Anaplasma platys*, as well as antigen of *Dirofilaria immitis.* Only the *Borrelia burgdorferi* and *Anaplasma* spp. results were included in the present analysis.

Data were collected from the IDEXX Reference Laboratories network and from veterinarians using IDEXX VetLab® Instrumentation and Software (IVLS). In addition to automated result capture from the IDEXX SNAPShot Dx® instrument, manual entry of visual results could be directly entered into the IVLS or recorded on the IDEXX SNAP Pro® instrument by hospital staff. Beginning in 2017, automated result interpretation was made available on the SNAP Pro with ProRead. Automated result interpretation is designed to mimic visual interpretation, and the results can be reviewed and revised by the hospital staff. The results used in this study were those approved and used by the veterinary practice. To ensure privacy, results were obtained without owner or clinic identifying information and thus repeat testing events cannot be excluded or accounted for in the analysis.

### Performance of test kits

Performance characteristics of the SNAP® 4Dx® and SNAP® 4Dx® Plus test kits have been reported previously [17, 18]. Briefly, the *B. burgdorferi* assay detects antibodies against the C_6_ peptide of *B. burgdorferi* with a sensitivity of 96.7%, a laboratory specificity of 100%, and a field specificity of 98.8% [19, 20]. Antibodies generated by past or current sub-clinical and clinical infections are identified, but antibodies due to canine vaccination are not detected [17].

The *Anaplasma* spp. assay uses a synthetic peptide of a major surface protein of *Anaplasma* spp. (MSP2/p44) to detect antibodies against *A. phagocytophilum* and *A. platys*. Reported sensitivity and specificity are 93.2% and 99.2%, respectively [19].

### Data and statistical analysis

All available results were collated by county, state, and year; information on individual practices or individual patients was not collected. Percent positive test results (canine seroprevalence) were calculated for each county, state, and year, by dividing the number of positive test results by the total number of the tests performed. Cumulative seroprevalence for each agent over the entire 8-year period was also calculated.

Data were curated to avoid introducing bias from low available test numbers or, for trend analysis, low overall seroprevalence consistent with non-endemic status. Prior to any analysis, results from individual counties with less than 30 tests performed in a single year and states with cumulative seroprevalence of < 0.5% for both agents or total test results < 50,000 were excluded. For the remaining 25 states, state-level trend analysis was performed only on states with > 1.0% cumulative prevalence for a given agent, while all counties with adequate numbers of test results were included in the county-level trend analysis.

The Cochran Armitage Trend test with total test count by year for weights was used to evaluate changes in annual seroprevalence within states and counties over the 8-year period from 2010–2017 using SAS v9.4 (SAS Institute Inc., Cary NC, USA). Due to the large number of data points available, thresholds for significance (α) were set at 0.0001 and 0.01 for states and counties, respectively. The Goodman Kruskal gamma test was also calculated to examine the direction and magnitude of trends. Trend lines were graphed with a linear regression model using Excel where m represents the slope (Microsoft Excel, Redmond, WA). Maps were constructed using MapViewer 8.0 (Golden Software, Golden, Colorado, USA).

## Results

After omitting counties and states with low number of tests, the remaining database consisted of 20,464,256 test results for *B. burgdorferi* and 19,901,123 test results for *Anaplasma* spp. over the 8-year period from 2010–2017. Data were available from 905/1431 counties (63.2%) in 25 states.

### *Borrelia burgdorferi*

#### State trends

**Fig. 1:**
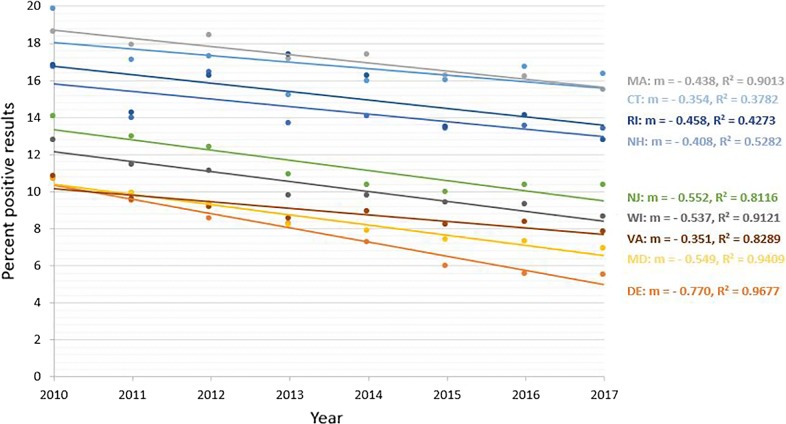
Decreasing trend in seroprevalence for antibodies to *Borrelia burgdorferi. Abbreviations*: CT, Connecticut; DE, Delaware; MA, Massachusetts; MD, Maryland; NH, New Hampshire; NJ, New Jersey; RI, Rhode Island; VA, Virginia; WI, Wisconsin

**Fig. 2:**
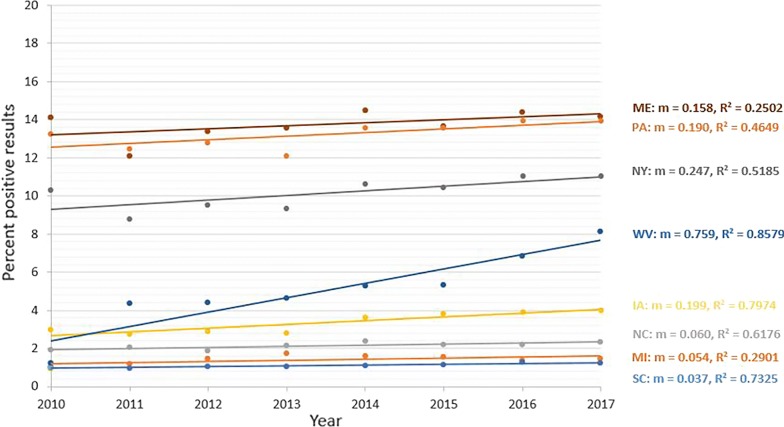
Increasing trend in seroprevalence for antibodies to *Borrelia burgdorferi. Abbreviations*: ME, Maine; NY, New York; PA, Pennsylvania; IA, Iowa; MI, Michigan; NC, North Carolina; SC, South Carolina; WV, West Virginia

Seroprevalence of antibodies to *Borrelia burgdorferi* from 9 states showed significant decreasing trend (*P* < 0.0001) over the 8-year period (Fig. 1). States with a decreasing trend included 7 in the Northeast (Connecticut, Delaware, Massachusetts, Maryland, New Hampshire, New Jersey and Rhode Island), one in the Southeast (Virginia), and one in the upper Midwest (Wisconsin) (Table 1). The relative percent change in annual seroprevalence in these states from 2010 to 2017 ranged from − 16.7% (Massachusetts) to − 48.6% (Delaware) (Table 1). Seroprevalence for *B. burgdorferi* from 8 states showed significant increasing trend (*P* < 0.0001) over the 8-year period (Fig. 2). States with an increasing trend included three in the Northeast (Maine, New York, and Pennsylvania), 3 in the Southeast (North Carolina, South Carolina, and West Virginia), and two in the Midwest (Iowa and Michigan) (Table 2). The relative percent change in annual seroprevalence in these states from 2010 to 2017 ranged from 0.7% (Maine) to 523.1% (West Virginia) (Table 2). A significant trend was not found for 6 states (Illinois, Indiana, Kentucky, Minnesota, North Dakota and Vermont), and trends in 2 states (Tennessee and Ohio) were not analyzed due to low (< 1.0%) state-wide cumulative percent prevalence over the 8-year study period.

**Table 1:** Percent positive test results (canine seroprevalence) for states with decreasing trend (*P* < 0.0001) in antibodies to *Borrelia burgdorferi*

State (*n*)^a^	Percent positive tests		Change (%)	Gamma (95% CI)^b^
	2010	2017		
Midwest				
Wisconsin (*n* = 999,422)	12.8	8.6	− 32.8	− 0.0811 (− 0.0854–− 0.0768)
Northeast				
Connecticut (*n* = 742,204)	19.9	16.3	− 18.1	− 0.0223 (− 0.0264–− 0.0182)
Delaware (*n* = 166,596)	10.7	5.5	− 48.6	− 0.1637 (− 0.1757–− 0.1517)
Massachusetts (*n* = 1,485,595)	18.6	15.5	− 16.7	− 0.0474 (− 0.0502–− 0.0446)
Maryland (*n* = 977,185)	10.8	6.9	− 36.1	− 0.1011 (− 0.1059–− 0.0963)
New Hampshire (*n* = 490,650)	16.7	13.4	− 19.7	− 0.0438 (− 0.0491–− 0.0385)
New Jersey (*n* = 927,468)	14.1	10.4	− 26.2	− 0.0754 (− 0.0797–− 0.0711)
Rhode Island (*n* = 150,452)	16.8	12.8	− 23.8	− 0.0574 (− 0.0667–− 0.0482)
Southeast				
Virginia (*n* = 1,508,270)	10.8	7.8	− 27.8	− 0.0522 (− 0.0560–− 0.0485)

^a^Total number of test results

^b^Gamma calculated for annual results for each state from 2010 through 2017, inclusive

**Table 2:** Percent positive test results (canine seroprevalence) for states with increasing trend (*P* < 0.0001) in antibodies to *Borrelia burgdorferi*

State (*n*)^a^	Percent positive tests		Change (%)	Gamma (95% CI)^b^
	2010	2017		
Midwest				
Iowa (*n* = 387,236)	3.0	4.0	33.3	0.0915 (0.0802–0.1028)
Michigan (*n* =1,137,522)	1.0	1.5	50.0	0.0137 (0.0037–0.0237)
Northeast				
Maine (*n* = 666,337)	14.1	14.2	0.7	0.0205 (0.0159–0.0261)
New York (*n* = 1,999,391)	10.3	11.1	7.8	0.0457 (0.0427–0.0487)
Pennsylvania (*n* = 1,955,701)	13.3	14.0	5.3	0.0278 (0.0251–0.0306)
Southeast				
North Carolina (*n* = 1,309,117)	1.9	2.3	21.1	0.0309 (0.0232–0.0387)
South Carolina (*n* = 344,547)	1.0	1.2	20.0	0.0510 (0.0300–0.0720)
West Virginia (*n* = 211,169)	1.3	8.1	523.1	0.2224 (0.2105–0.2343)

^a^Total number of test results

^b^Gamma calculated for annual results for each state from 2010 through 2017, inclusive

#### County trends

**Fig. 3:**
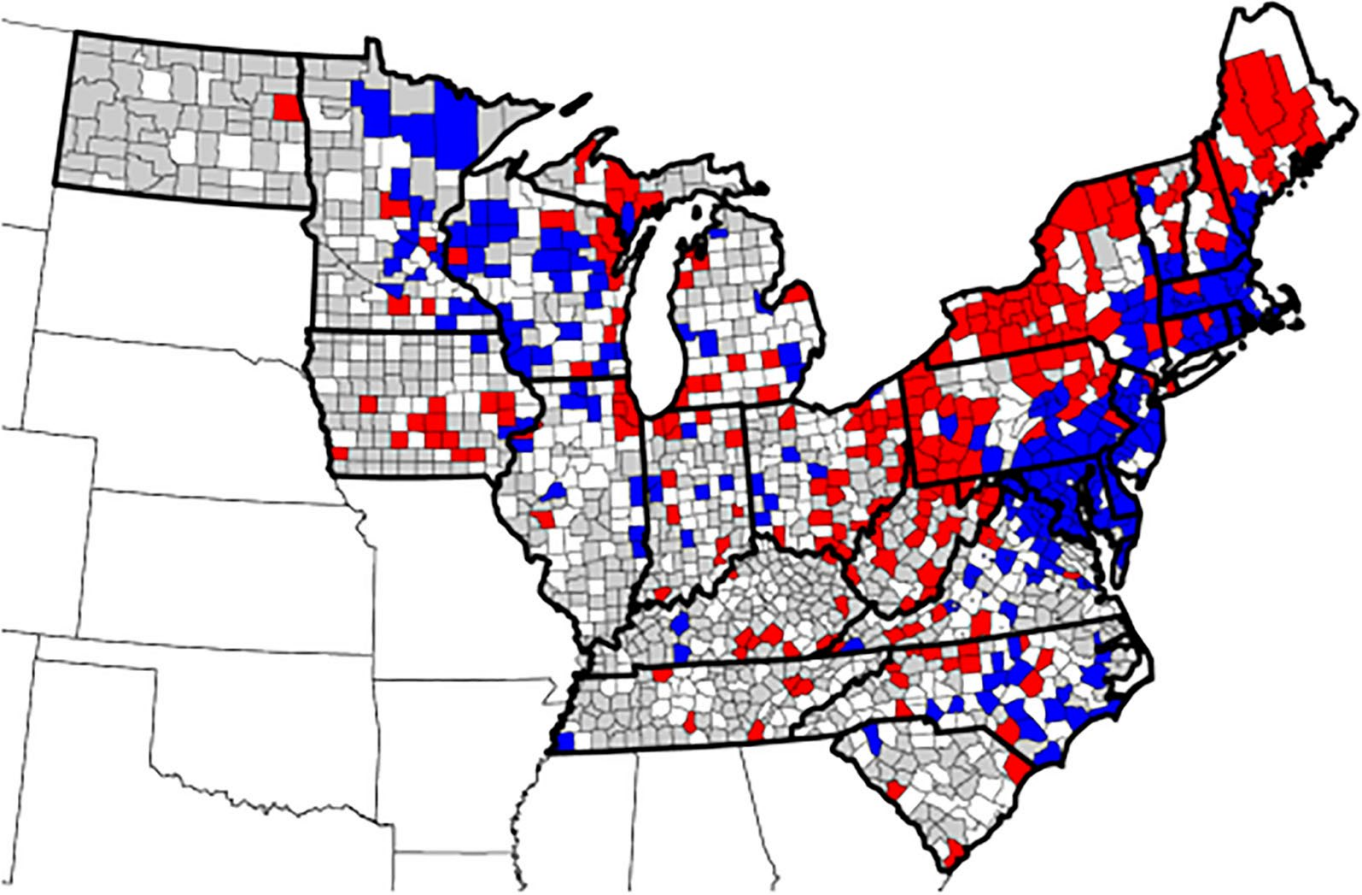
County trends in seroprevalence for antibodies to *Borrelia burgdorferi*, 2010–2017. Trends evident included decreasing (blue), increasing (red), and no significant change (white). Counties for which adequate data were not available are shown in gray

Seroprevalence for *B. burgdorferi* by individual counties also showed significant trends (*P* < 0.01). Of the 905 counties for which adequate data were available, 215/905 (23.8%) showed increasing trends in seroprevalence, 217/905 (24.0%) showed decreasing trends, and 473/905 (52.3%) showed no significant change (Fig. 3). County trend analysis identified states in which seroprevalence in a majority of counties was increasing (*n* = 2, New York and West Virginia) or decreasing (*n* = 6, Connecticut, Delaware, Massachusetts, Maryland, New Jersey and Rhode Island). Mixed county trends, in which some, but not a majority, of counties were increasing or decreasing, were evident in the remaining 17 states (Table 3). Mapping trends by county revealed geographical areas of increase or decrease in several states (Fig. 3).

**Table 3:** County trends (*P* < 0.01) by state in seroprevalence of antibodies to *Borrelia burgdorferi*

State (*n*)^a^	Counties with decreasing trend *n* (%)	Counties with increasing trend *n* (%)	Counties with no change *n* (%)
Majority counties decreasing			
Connecticut (*n* = 8)	**5 (62.5)**	2 (25.0)	1 (12.5)
Delaware (*n* = 3)	**3 (100.0)**	0	0
Massachusetts (*n* = 14)	**11 (78.6)**	1 (7.1)	2 (14.3)
Maryland (*n* = 23)	**19 (82.6)**	1 (4.3)	3 (13.0)
New Jersey (*n* = 21)	**14 (66.7)**	1 (4.8)	6 (28.6)
Rhode Island (*n* = 5)	**4 (80.0)**	0	1 (20.0)
Majority counties increasing			
New York (*n* = 60)	9 (15.0)	**32 (53.3)**	19 (31.7)
West Virginia (*n* = 33)	3 (9.1)	**21 (63.6)**	9 (27.3)
Mixed county trends			
Iowa (*n* = 43)	3 (7.0)	13 (30.2)	27 (63.0)
Illinois (*n* = 54)	8 (14.0)	6 (11.0)	40 (74.0)
Indiana (*n* = 52)	6 (11.5)	11 (21.2)	35 (67.3)
Kentucky (*n* = 41)	2 (4.9)	9 (22.0)	30 (73.2)
Maine (*n* = 16)	2 (12.5)	7 (43.8)	7 (43.8)
Michigan (*n* = 60)	7 (11.7)	14 (23.3)	39 (65.0)
Minnesota (*n* = 47)	15 (31.9)	3 (6.4)	29 (61.7)
North Carolina (*n* = 70)	17 (24.3)	12 (17.1)	41 (58.6)
North Dakota (*n* = 8)	0	1 (12.5)	7 (87.5)
New Hampshire (*n* = 10)	3 (30.0)	4 (40.0)	3 (30.0)
Ohio (*n* = 60)	6 (10.0)	22 (36.7)	32 (53.3)
Pennsylvania (*n* = 61)	20 (32.8)	27 (44.3)	14 (23.0)
South Carolina (*n* = 23)	1 (4.3)	3 (13.0)	19 (82.6)
Tennessee (*n* = 37)	1 (2.7)	5 (13.5)	31 (83.8)
Virginia (*n* = 86)	31 (36.0)	8 (9.3)	47 (54.7)
Vermont (*n* = 12)	3 (25.0)	4 (33.3)	5 (41.7)
Wisconsin (*n* = 58)	24 (41.4)	8 (13.8)	26 (44.8)

^a^Number of counties evaluated; data not available from every county in every state listed

*Note*: Numbers in boldface indicate > 50% of counties evaluated in that state had an increasing or decreasing trend (*P* < 0.01)

### *Anaplasma phagocytophilum*

#### State trends

**Fig. 4:**
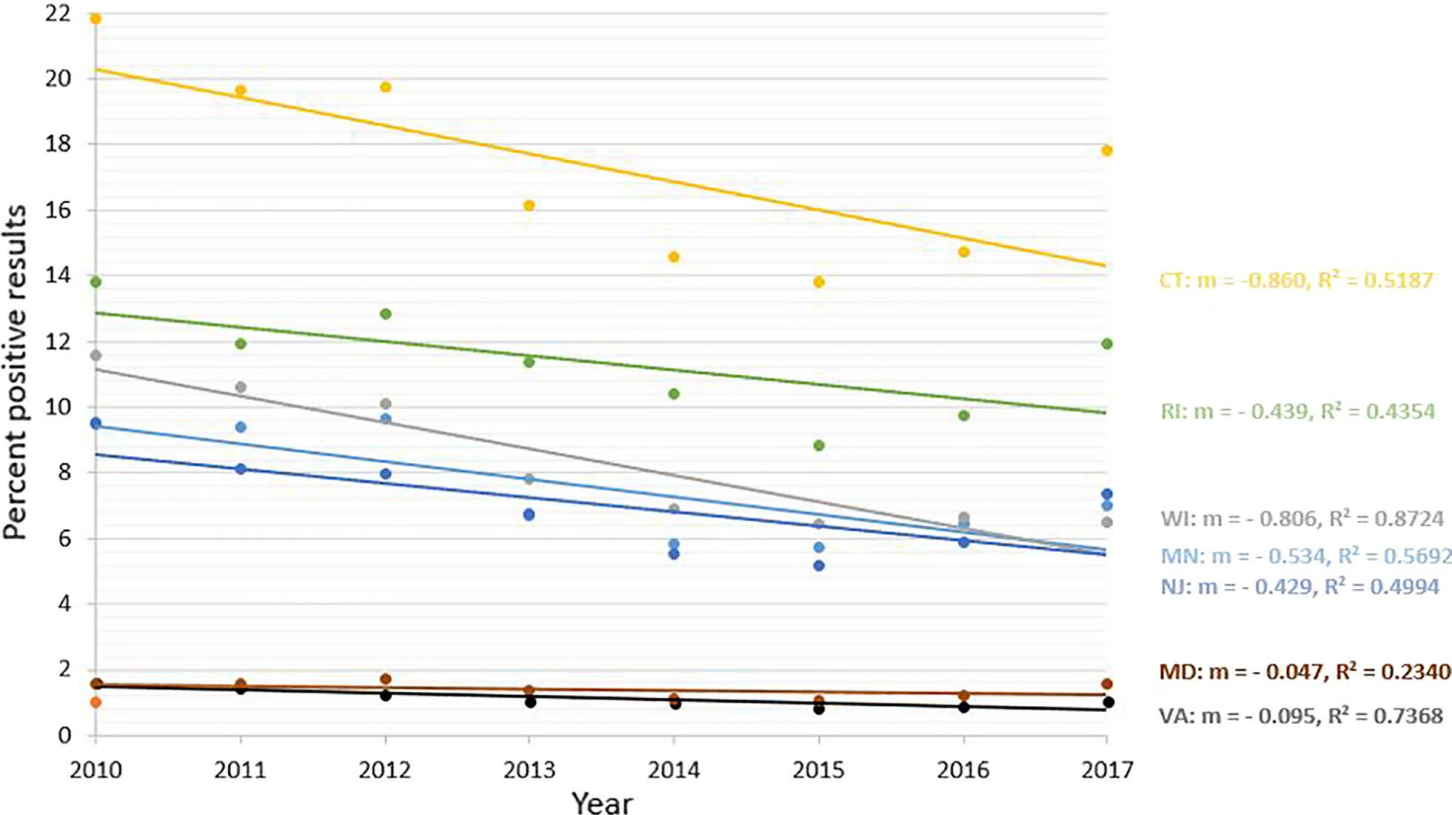
Decreasing trend in seroprevalence for antibodies to *Anaplasma phagocytophilum. Abbreviations*: CT, Connecticut; MD, Maryland; NJ, New Jersey; RI, Rhode Island; VA, Virginia; MN, Minnesota; WI, Wisconsin

**Fig. 5:**
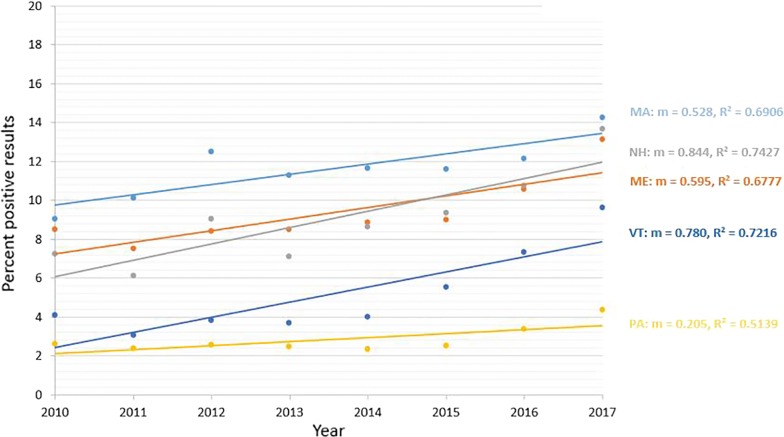
Increasing trend in seroprevalence for antibodies to *Anaplasma phagocytophilum. Abbreviations*: MA, Massachusetts; ME, Maine; NH, New Hampshire; PA, Pennsylvania; VT, Vermont

Seroprevalence for antibodies to *A. phagocytophilum* from 7 states showed significant decreasing trend (*P* < 0.0001) over the 8-year period (Fig. 4). States with a decreasing trend included 4 in the Northeast (Connecticut, Maryland, New Jersey and Rhode Island), one in the Southeast (Virginia), and two in the Midwest (Minnesota and Wisconsin) (Table 4). The relative percent change in annual seroprevalence in these states from 2010 to 2017 in these states ranged from − 13.8% (Rhode Island) to − 44.0% (Wisconsin) (Table 3). Seroprevalence for *A. phagocytophilum* from 5 states showed significant increasing trend (*P* < 0.0001) over the 8-year period (Fig. 5). All states with an increasing trend were in the Northeast (Maine, Massachusetts, New Hampshire, Pennsylvania and Vermont) (Table 4). The relative percent change in annual seroprevalence in these states from 2010 to 2017 ranged from 55.3% (Maine) to 134.2% (Vermont) (Table 5). A significant trend was not found for 2 states (New York and North Dakota), and trends in 11 states (Delaware, Iowa, Illinois, Indiana, Kentucky, Michigan, North Carolina, Ohio, South Carolina, Tennessee and West Virginia) were not analyzed due to low (< 1.0%) state-wide cumulative percent prevalence over the 8-year study period.

**Table 4:** Percent positive test results (canine seroprevalence) for states with decreasing trend (*P* < 0.0001) in antibodies to *Anaplasma phagocytophilum*

State (*n*)^a^	Percent positive tests		Change (%)	Gamma (95% CI)^b^
	2010	2017		
Midwest				
Minnesota (*n* = 823,260)	9.6	7.0	− 27.1	− 0.0944 (− 0.1001–− 0.0888)
Wisconsin (*n* = 999,400)	11.6	6.5	− 44.0	− 0.1457 (− 0.1505–− 0.1409)
Northeast				
Connecticut (*n* = 741,964)	21.8	17.8	− 18.3	− 0.0545 (− 0.0587–− 0.0503)
Maryland (*n* = 937,104)	1.6	1.6	0	− 0.0240 (− 0.0361–− 0.0119)
New Jersey (*n* = 918,355)	9.5	7.3	− 23.2	− 0.0751 (− 0.0807–− 0.0696)
Rhode Island (*n* = 144,637)	13.8	11.9	− 13.8	− 0.0622 (− 0.0732–− 0.0512)
Southeast				
Virginia (*n* = 1,466,464)	1.6	1.1	− 31.2	− 0.0851 (− 0.0961–− 0.0741)

^a^Total number of test results

^b^Gamma calculated for annual results for each state from 2010 through 2017, inclusive

**Table 5:** Percent positive test results (canine seroprevalence) for states with increasing trend (*P* < 0.0001) in antibodies to *Anaplasma phagocytophilum*

State (*n*)^a^	Percent positive tests		Change (%)	Gamma (95% CI)^b^
	2010	2017		
Northeast				
Massachusetts (*n* = 1,464,062)	9.1	14.3	57.1	0.0759 (0.0726–0.0792)
Maine (*n* = 660,847)	8.5	13.2	55.3	0.1111 (0.1056–0.1167)
New Hampshire (*n* = 485,522)	7.3	13.7	87.7	0.1614 (0.1550–0.1678)
Pennsylvania (*n* = 1,911,290)	2.6	4.4	69.2	0.1377 (0.1320–0.1435)
Vermont (*n* = 197,082)	4.1	9.6	134.2	0.2601 (0.2474–0.2729)

^a^Total number of test results

^b^Gamma calculated for annual results for each state from 2010 through 2017, inclusive

#### County trends

**Fig. 6:**
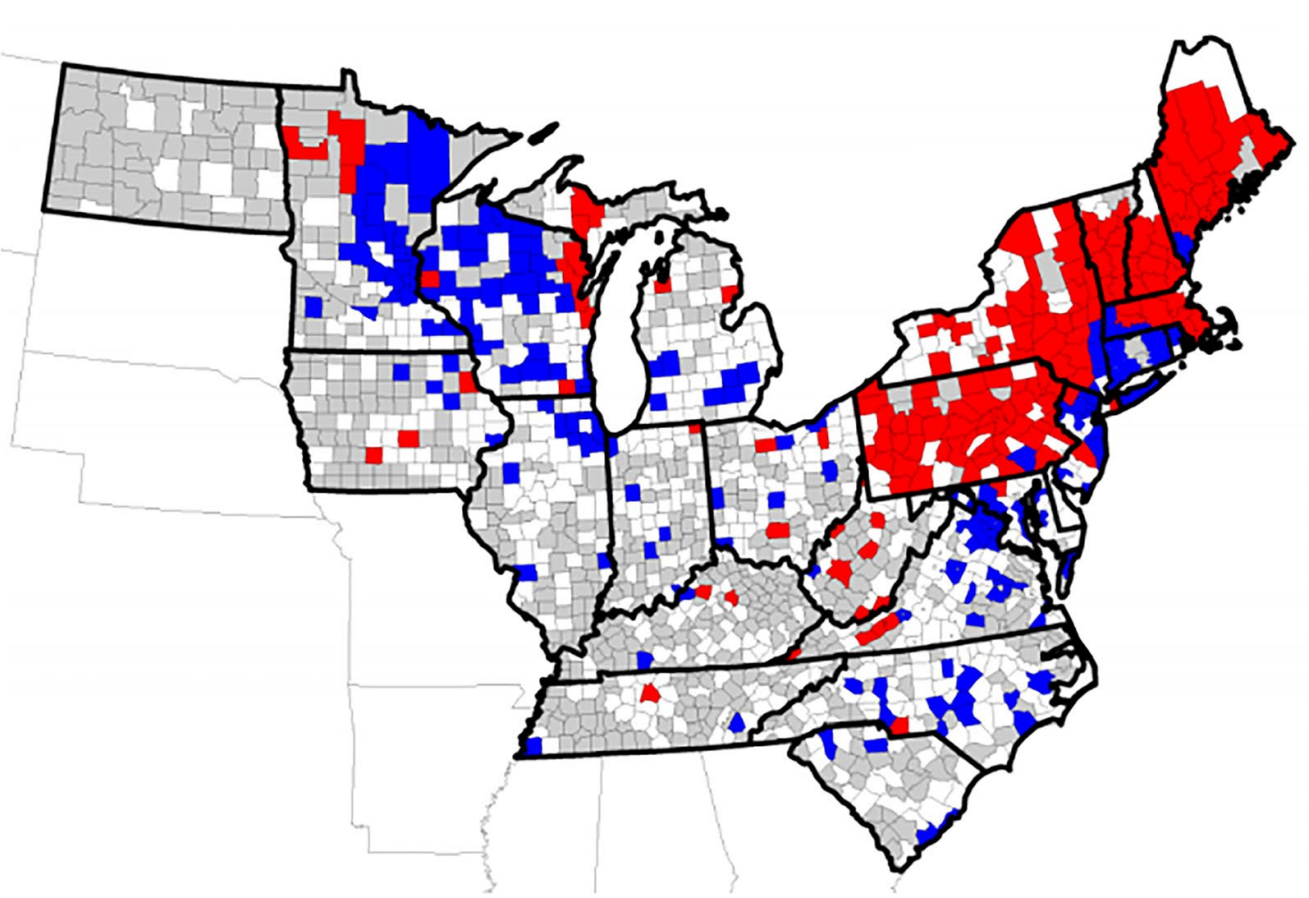
County trends in seroprevalence for antibodies to *Anaplasma phagocytophilum*, 2010–2017. Trends evident included decreasing (blue), increasing (red), and no significant change (white). Counties for which adequate data were not available are shown in gray

Seroprevalence for *A. phagocytophilum* by individual counties also showed significant trends (*P* < 0.01). Of the 887 counties for which adequate data were available, 157/887 (17.7%) showed increasing trends in seroprevalence, 167/887 (18.8%) showed decreasing trends, and 563/887 (63.5%) showed no significant change (Fig. 6). County trend analysis identified states in which seroprevalence in a majority of counties was increasing (*n* = 5, Maine, Massachusetts, New Hampshire, Pennsylvania and Vermont) or decreasing (*n* = 3, Connecticut, New Jersey and Wisconsin), while mixed county trends were evident in the remaining 17 states (Table 6). Mapping trends by county revealed focused areas of increase or decrease in several states (Fig. 6).

**Table 6:** County trends (*P* < 0.01) by state in seroprevalence of antibodies to *Anaplasma phagocytophilum*

State (*n*)^a^	Counties with decreasing trend *n* (%)	Counties with increasing trend *n* (%)	Counties with no change *n* (%)
Majority counties decreasing			
Connecticut (*n* = 8)	**6 (75.0)**	0 (0.0)	2 (25.0)
New Jersey (*n* = 21)	**12 (57.1)**	2 (9.5)	7 (33.3)
Wisconsin (*n* = 58)	**29 (50.0)**	6 (10.3)	23 (39.6)
Majority counties increasing			
Massachusetts (*n* = 14)	3 (21.4)	**8 (57.1)**	3 (21.4)
Maine (*n* = 16)	1 (6.2)	**14 (87.5)**	1 (6.2)
New Hampshire (*n* = 10)	0 (0.0)	**9 (90.0)**	1 (10.0)
Pennsylvania (*n* = 61)	1 (1.6)	**47 (77.0)**	13 (21.3)
Vermont (*n* = 11)	0 (0.0)	**9 (81.8)**	2 (18.2)
Mixed county trends			
Delaware (*n* = 3)	0 (0.0)	0 (0.0)	3 (100)
Iowa (*n* = 42)	5 (11.9)	3 (7.1)	34 (80.9)
Illinois (*n* = 50)	9 (18.0)	0 (0.0)	41 (82.0)
Indiana (*n* = 48)	4 (8.3)	1 (2.0)	43 (89.5)
Kentucky (*n* = 41)	2 (4.9)	2 (4.9)	37 (90.2)
Maryland (*n* = 23)	6 (26.1)	1 (4.3)	16 (69.6)
Michigan (*n* = 60)	7 (11.7)	4 (6.7)	49 (81.7)
Minnesota (*n* = 47)	23 (48.9)	3 (6.4)	21 (44.7)
North Carolina (*n* = 69)	12 (17.4)	1 (1.4)	56 (81.1)
North Dakota (*n* = 8)	0 (0.0)	0 (0.0)	8 (100)
New York (*n* = 60)	6 (10.0)	29 (48.3)	25 (41.7)
Ohio (*n* = 58)	7 (12.1)	3 (5.1)	48 (82.7)
Rhode Island (*n* = 5)	2 (40.0)	1 (20.0)	2 (40.0)
South Carolina (*n* = 22)	3 (13.6)	0 (0.0)	19 (86.4)
Tennessee (*n* = 36)	2 (5.5)	1 (2.8)	33 (91.7)
Virginia (*n* = 85)	24 (28.2)	5 (5.9)	56 (65.9)
West Virginia (*n* = 31)	3 (9.6)	8 (25.8)	20 (64.5)

^a^Number of counties evaluated; data not available from every county in every state listed

*Note*: Numbers in boldface indicate > 50% of counties evaluated in that state had an increasing or decreasing trend (*P* < 0.01)

## Discussion

Lyme borreliosis (LB) and anaplasmosis (AN) are common among people and dogs in much of the eastern USA, and several studies document a growing risk of infection concomitant with increasing tick populations and continued expansion of the geographical area where these pathogens are known to be transmitted [4, 21–24]. Canine serology has been shown to be an indicator of human disease risk in a given region [13, 14, 25]. Our analysis of canine serologic data confirmed increasing trends in seroprevalence of *Bb* and *Anaplasma* spp. in several areas, including northern New England, upstate New York, and western Pennsylvania, as well as in some southern states where the *I. scapularis-*pathogen-reservoir host maintenance cycles necessary to maintain these agents have apparently only recently become established [26, 27]. Interestingly, the data in the present study also documented for the first time decreasing trends in canine seroprevalence for these tick-borne agents in some regions, including in several Mid-Atlantic States and in the Midwest where autochthonous transmission of *Bb* and *Ap* has long been recognized. Indeed, data from the present study suggest that state-wide prevalence of antibodies to *Bb* in dogs may have decreased by as much as 25–50% in some states over the last decade (Table 1, Fig. 1).

Lyme borreliosis was first recognized in Connecticut in the 1970s, and risk of infection in that state remains high, with more than 20,000 new human cases reported to the Connecticut Department of Public Health from 2007 to 2017 [28–30]. However, passive tick surveillance in the state over the same time span documented a decrease in both the rate of *I. scapularis* nymphs submitted and the prevalence of *Bb* infection in those nymphs [30], and total annual human case reports in Connecticut decreased from 3058 in 2007 to 2051 in 2017 [2, 3]. Although multiple factors influence reports of human cases of a given disease, the decreasing trend in canine seroprevalence identified in the present study is consistent with the trend reported by others of stable to decreasing human case reports in many high incidence states [31]. However, continued field research in the region is necessary to determine if the intensity of the tick population or prevalence of infection in questing ticks is declining in concert with canine seroprevalence and human case reports.

In contrast, risk of LB appears to be growing in other states in the northeastern USA. For example, in Maine, *Bb* transmission was first documented in the southern part of the state, but as of 2014, locally acquired cases had been diagnosed in all 16 counties in the state and the number of cases reported each year continues to rise [32], with 751 cases reported in 2010 and 1850 cases reported in 2017 [2, 3]. *Ixodes scapularis* was first recognized in Maine in 1988; over the next 18 years, statewide surveillance showed that cases of LB at the county level were closely related to submissions of *I. scapularis* nymphs [33]. The county trend analysis in the present paper supports the interpretation that transmission risk has continued to increase in Maine in the last decade although again, continued field surveillance of *Bb* prevalence in ticks is necessary to confirm this explanation for the increasing trends seen.

Clustered trends on a county basis also appeared in our analysis and were particularly evident in states where continued geographical expansion of *Bb* transmission has been recognized in the last decade, including western New York and western Pennsylvania. Interestingly, the analyses in the present paper revealed increasing trends in canine seroprevalence to *Bb* in western Pennsylvania (Fig. 3), consistent with other recent publications documenting transmission of *Bb* and subsequent human disease state-wide in Pennsylvania [34]. However, the present study also documented decreasing trends in canine seroprevalence to *Bb* in southeastern Pennsylvania where the pathogen has long been transmitted, a shift that is less well understood but may be related to the declining trends seen in the Mid-Atlantic States.

Counties with significant increasing trends in canine seroprevalence to *Bb* were also evident in some states where overall canine seroprevalence remains low. For example, several counties in Kentucky and Tennessee showed significant increasing trends in the present study, with 5–20% of dogs in affected counties testing positive despite the fact that statewide, only 1.3% and 0.8%, respectively, of dogs tested positive, supporting the interpretation that autochthonous transmission of *Bb* may now be occurring in focused areas in these two states. This supposition is supported by the fact that wild caught *I. scapularis* ticks infected with *Bb* were reported from Kentucky and the upper Tennessee Valley for the first time in ticks collected from 2015–2017 and in 2017, respectively [26, 35].

Human AN was initially described from northern Minnesota and Wisconsin in 1994 [36]; the annual number of reported cases from those two states increased from 79 in 2000 to 1217 in 2010, but has since stabilized somewhat, with an average of 1312 cases (995–1504) reported each year from 2011 to 2017 [2, 3, 37]. This leveling in the number of cases reported annually may be due, in part, to underreporting in some highly endemic areas [38]. However, while disease caused by *Ap* remains endemic in these two states, the finding in the present paper of decreasing trends in canine seroprevalence for antibodies to *Anaplasma* spp. in both Minnesota and Wisconsin suggests overall risk of *Ap* infection could be decreasing in that region. One study found that *Ap* infection prevalence in *I. scapularis* nymphs decreased from 15.8% to 7.7% in ticks collected from southeastern Wisconsin from 2009 to 2013 although a similar decrease was not seen in ticks collected from central Wisconsin [39].

Increasing state-wide trends in canine seroprevalence for *Anaplasma* spp. were also noted in the present study and mirror increases evident in human disease reports. For example, in Vermont the canine prevalence of antibodies to *Anaplasma* spp. increased from 4.1% to 9.6% over the 8 years considered in the present study (Table 5). Similarly, annual reports to CDC of human AN in Vermont increased from none in 2010 to almost 400 cases in 2017, and *I. scapularis* ticks were confirmed to be present in 78.6% of counties in Vermont by 2015 [2, 3, 40, 41]. Additional assessment of questing ticks for prevalence of *Ap* infection in the states where canine serology is significantly changing (Fig. 6) is needed to confirm these observations and may help determine the degree to which risk of infection is shifting.

The present study used canine serology to document trends in infection risk with *Bb* and *Anaplasma* spp. in different geographies and identified both increasing and decreasing trends. The reasons for increasing trends are unknown, but they may be due to expansion of *I. scapularis* populations that is thought to be primarily driven by habitat change, increased reservoir host populations, and climate change [13, 42]. *Ixodes scapularis* are dependent upon availability of host populations and thus supported by processes such as increased deer populations or reforestation [43]. Tick populations also respond to abiotic conditions such as temperature and humidity. The climate present at higher elevations and latitudes historically provided poor tick habitat as the lower temperatures and humidity found in those locations restricted survival of *I. scapularis*, particularly the immature stages [44–46], but recent shifts in climate have resulted in increased temperature and humidity at higher altitudes and latitudes, allowing expansion and survival of tick populations to these new locales. As predicted, such changes have apparently resulted in the establishment of LB in southern Ontario, Quebec, and Nova Scotia in recent years and may also be contributing to increasing trends in seroprevalence in more northern regions of North America as well as at higher elevations in southern Appalachia [42, 47–49].

Similarly, the reasons for the decreasing trends evident in the Mid-Atlantic States cannot be determined from the present study, but as temperatures increase above those readily tolerated by *I. scapularis* at lower elevations and latitudes, tick populations could be adversely affected or tick phenology may shift, potentially reducing transmission of infection [50]. *Ixodes scapularis* in the southern USA exhibits a dramatically different phenology and questing behavior than that seen in populations of this tick in the northern US, a variation that has been attributed, in part, to an adaptation that may facilitate tick survival in higher temperature regions [50–53]. Additional research will be needed to directly assess the effect of climate change on tick populations, if any, in this region and to evaluate the contribution such an effect may have on the apparent serologic trends evident in the present paper.

One alternative explanation for the decreasing trends seen in some regions in the present study is the potential influence of tick control and vaccination against *Bb* in pet dogs [54–56], both of which reduce canine infection. Systemic isoxazolines were first introduced in the USA in 2014 and have been shown to both kill *I. scapularis* and reduce transmission of *Bb* and *Ap* to dogs [56]. Canine vaccination against *Bb,* which is widely practiced in endemic areas, does not generate antibodies that are detected on the serologic test used in the present study, but it does limit infection and thus would be expected to reduce seroprevalence over time [17, 54, 55]. Unfortunately, we do not know if or to what extent use of these prevention practices in dogs differs regionally in a way that may have influenced results. Vaccines to prevent *Bb* infection in people are not currently available in the USA and so we would not expect such interventions to similarly influence reports of human disease which are also declining in some areas [31].

Limitations of the present study include incomplete clinical information on the source of the data (dogs presenting to veterinary practices), the unknown history of the dogs themselves, and the scope of pathogens detected by the assays used. Unfortunately, we do not know the clinical rationale veterinarians had for testing each of the dogs that had results included in the analysis. Routine, annual screening of clinically normal dogs for tick-borne agents to facilitate early detection and treatment is recommended by advisory groups such as the American College of Veterinary Internal Medicine [9], but targeted testing of sick dogs also occurs and may have influenced the outcome. Prevalence of vector-borne infections also are likely to be affected by the age of the dogs tested, lifestyle and tick exposure risk, and overall health status, but that information was not available for inclusion in the analysis. In addition, although the assays used were designed to detect antibodies to *Bb* and *Anaplasma* spp., these tests may perform differently when used to test dogs infected with newly emergent, related organisms or those infected with agents undergoing antigenic variation [57, 58].

## Conclusions

Evaluation of trends in canine *Bb* and *Anaplasma* spp. seroprevalence in the present study revealed evidence for continued geographical expansion of these agents and their corresponding natural maintenance cycles into new areas in the USA. However, we also identified trends suggesting canine infections may be declining in some regions where these agents have long been present. Although not evaluated in the present study, these downward trends in canine seroprevalence could be the result of overall decreased infection risk together with enhanced veterinary care employing a combination of canine vaccination against *Bb* infection, tick control, and regular screening for vector-transmitted infections. Nonetheless, while infection pressure intensity appears to be shifting geographically, risk of LB and AN remains high in regions of North America with dense populations of infected *I. scapularis* [13, 42]. Monitoring trends in canine seroprevalence to *Bb* and *Anaplasma* spp. allows veterinary and public health to target education efforts toward areas where these infections are only recently recognized as well as regions where they have been long established.

## Data Availability

The summary datasets analyzed in the present study are available from the corresponding author and IDEXX Laboratories, Inc. on reasonable request.

## Abbreviations

AN: anaplasmosis
*Ap*: *Anaplasma phagocytophilum*
*Bb*: *Borrelia burgdorferi* (*sensu stricto*)
CDC: Centers for Disease Control and Prevention
IVLS: IDEXX VetLab® Instrumentation and Software
LB: Lyme borreliosis
MSP: major surface protein
